# Glucose transporter isoform 1 expression enhances metastasis of malignant melanoma cells

**DOI:** 10.18632/oncotarget.4977

**Published:** 2015-07-22

**Authors:** Andreas Koch, Sven Arke Lang, Peter Johannes Wild, Susanne Gantner, Abdo Mahli, Gerrit Spanier, Mark Berneburg, Martina Müller, Anja Katrin Bosserhoff, Claus Hellerbrand

**Affiliations:** ^1^ Department of Internal Medicine I, University Hospital Regensburg, Germany; ^2^ Department of Surgery, University Hospital Regensburg, Germany; ^3^ Institute of Pathology, University Hospital Zurich, Switzerland; ^4^ Department of Dermatology, University Hospital Regensburg, Germany; ^5^ Department of Cranio-Maxillo-Facial Surgery, University Hospital Regensburg, Germany; ^6^ Institute of Biochemistry, University of Erlangen, Germany

**Keywords:** GLUT1, melanoma, metastasis, glycolysis, JNK

## Abstract

The glucose transporter isoform 1 (GLUT1; SLC2A1) is a key rate-limiting factor in the transport of glucose into cancer cells. Enhanced GLUT1 expression and accelerated glycolysis have been found to promote aggressive growth in a range of tumor entities. However, it was unknown whether GLUT1 directly impacts metastasis. Here, we aimed at analyzing the expression and function of GLUT1 in malignant melanoma. Immunohistochemical analysis of 78 primary human melanomas on a tissue micro array showed that GLUT1 expression significantly correlated with the mitotic activity and a poor survival. To determine the functional role of GLUT1 in melanoma, we stably suppressed GLUT1 in the murine melanoma cell line B16 with shRNA. GLUT1 suppressed melanoma cells revealed significantly reduced proliferation, apoptosis resistance, migratory activity and matrix metalloproteinase 2 (MMP2) expression. In a syngeneic murine model of hepatic metastasis, GLUT1-suppressed cells formed significantly less metastases and showed increased apoptosis compared to metastases formed by control cells. Treatment of four different human melanoma cell lines with a pharmacological GLUT1 inhibitor caused a dose-dependent reduction of proliferation, apoptosis resistance, migratory activity and MMP2 expression. Analysis of MAPK signal pathways showed that GLUT1 inhibition significantly decreased JNK activation, which regulates a wide range of targets in the metastatic cascade. In summary, our study provides functional evidence that enhanced GLUT1 expression in melanoma cells favors their metastatic behavior. These findings specify GLUT1 as an attractive therapeutic target and prognostic marker for this highly aggressive tumor.

## INTRODUCTION

Melanoma is the most aggressive form of skin cancer. Even very thin primary tumors may seed metastases and precipitate rapid death [1, 2]. The worldwide incidence of melanoma is increasing more than in any other neoplastic disease [3]. Still, to date there is no effective therapy for metastatic melanoma and at the molecular level the disease progression is poorly understood [4].

In the 1920s, Otto Warburg made the observation that tumor cells utilize glycolysis instead of mitochondrial oxidative phosphorylation for energy production even under oxygen-rich conditions. Lately, the “Warburg effect” has experienced a revival, because it has been shown that aerobic glycolysis governs tumor cell biology [5, 6].

The glucose transporter isoform 1 (GLUT1; SLC2A1) is a key rate-limiting factor in the transport and metabolism of glucose in cancer cells. GLUT1 expression is primarily undetectable in normal epithelial tissues and benign epithelial tumors. However, GLUT1 is overexpressed in a significant proportion of human carcinomas [7-9]. The apparent expression of a certain type of glucose transporter suggests an important role for this transporter in tumor biology. Therefore, it has been hypothesized that elevated GLUT1 expression by human carcinomas indicates enhanced utilization of energy and an increased metabolic state. This may induce proliferation and tumor growth and herewith indirectly promote metastatic behavior. Actually, GLUT1 protein expression confers poor prognosis in a wide range of solid tumors [10-14] but experimental studies which functionally proof an impact of GLUT1 expression on metastasis are missing. Furthermore, malignant melanoma studies regarding GLUT1 expression have revealed inconclusive results and the biological significance of GLUT1 in disease progression has remained unknown [15-19].

Here, we aimed to analyze GLUT1 expression in a large series of benign nevi, primary melanomas and melanoma metastases and correlated GLUT1 expression levels with clinicopathological characteristics. Furthermore, we analyzed the functional effect of GLUT1 expression on growth and metastasis of melanoma cells *in vitro* and *in vivo*. Together, experimental and patients data show that enhanced GLUT1 expression advances both growth and metastasis of malignant melanoma.

## RESULTS

### GLUT1 expression in primary human melanoma and melanoma metastases

**Figure 1:**
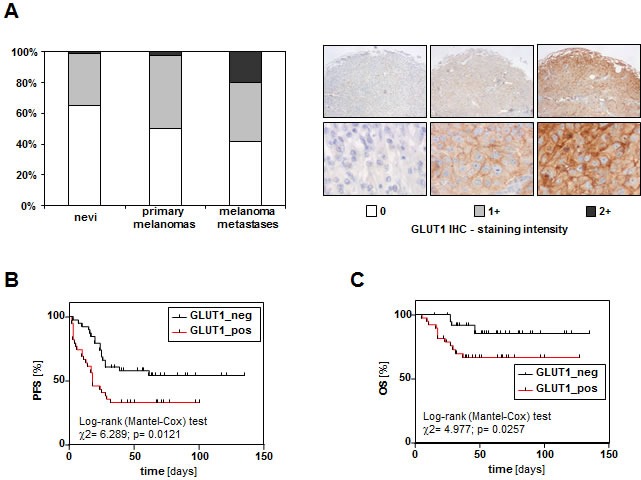
GLUT1 expression in human nevi, primary malignant melanomas and melanoma metastases GLUT1 immunohistochemical staining was performed on a TMA comprising 123 benign nevi, 78 primary human malignant melanomas and 60 melanoma metastases. Staining intensity was categorized into absent (0), weak (1+) and strong (2+). **A.** Representative images of primary tumors with the 3 different GLUT1 staining intensities are depicted in the right panel. Percentage of GLUT1 staining intensities in nevi, primary malignant melanomas and melanoma metastases (left panel). Kaplan-Meier tables showing **B.** progression free survival (PFS) and **C.** overall survival (OS) of melanoma patients with GLUT1 negative primary tumors (*n* = 39) and patients with GLUT1 positive (staining intensity 1+ or 2+) primary tumors (*n* = 39).

First, we analyzed GLUT1 expression in a series of 118 benign nevi, 78 primary human malignant melanomas, and 60 melanoma metastases applying GLUT1 immunohistochemistry and tissue microarray (TMA) technology [20]. In 65% (77/118) of the nevi no GLUT1 immunosignal was detectable, 35% (41/118) showed weak GLUT1 staining and in only one nevus strong GLUT1 staining was detectable (Figure 1A). In contrast, in only 50% (39/78) of primary melanoma tissues no GLUT1 immunosignal was detectable, 47% (37/78) showed at least weak staining and 2 tissues had a strong GLUT1 immunosignal (*p* = 0.038 compared to nevi) (Figure 1A). Melanoma metastases revealed an even stronger GLUT1 immunosignal compared to primary melanomas (*p* = 0.004). In only 42% (29/69) of metastases no GLUT1 immunosignal was detectable but 20% (12/69) showed strong GLUT1 staining (Figure 1A). For descriptive data analysis, clinico-pathological characteristics of primary tumors were compared with GLUT1 immunohistochemistry (Table 1). Interestingly, GLUT1 expression significantly correlated with the proliferation rate (KI67 labeling index) of primary malignant melanomas. No correlation was found between GLUT1 expression and age and gender of melanoma patients or the Clark level, thickness, and the growth pattern of the primary tumors. Importantly, patients with GLUT1 positive tumors revealed a significantly lower progression free (PFS) and overall survival (OS) (Figure 1B, 1C). Together, these data indicate that GLUT1 expression correlates with metastasis and a poor prognosis in patients with malignant melanoma.

**Table 1:** Clinico-pathologic parameters in relation to GLUT1 immunohistochemistry (IHC)

			GLUT1 IHC			
Variable	Categorization	analyzable (n)	0 (n)	1+ (n)	2+ (n)	P^1^
Primary malignant melanomas^2^						
Age at diagnosis						
	≤60 years	46	24	21	1	0.910
	>60 years	32	15	16	1	
Gender						
	female	41	16	24	1	0.074
	male	37	23	13	1	
Clark level^3^						
	I	1	0	1	0	
	II	1	1	0	0	
	III	10	4	6	0	0.930
	IV	50	25	23	2	
	V	15	8	7	0	
Tumor thickness						
	≤2.0mm	24	15	8	1	0.193
	>2.0mm	54	24	29	1	
Growth pattern^4^						
	NOS	13	6	7	0	
	SSM	32	18	13	1	
	ALM	5	2	3	0	0.940
	NMM	27	13	13	1	
	LMM	1	0	1	0	
Ki67 labeling index						
	≤5%	39	26	13	0	**0.019**
	>5%	22	8	12	2	

1 Fisher's exact test (two-sided), bold face representing significant data;

2 only initial and unifocal malignant melanomas were included;

3 according to UICC: TNM Classification of Malignant Tumours. 6th edn (2002), Sobin LH, Wittekind CH (eds.) Wiley, New York;

4 NOS, not otherwise specified; SSM, superfical spreading melanoma; ALM, acral lentiginous melanoma; NMM, nodular malignant melanoma; LMM, lentigo maligna melanoma.

### Inhibition of GLUT1 expression in murine B16 melanoma cells

**Figure 2:**
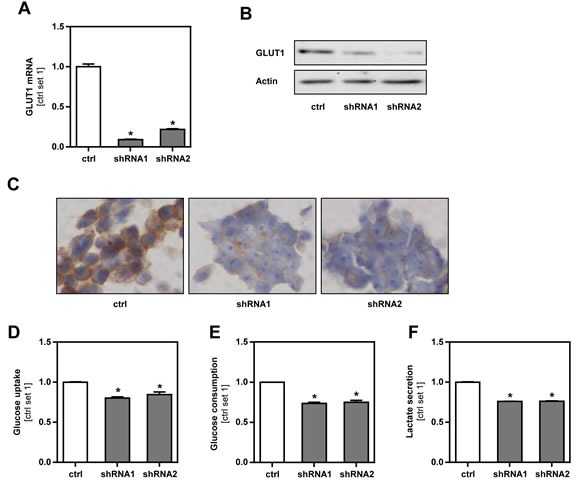
Inhibition of GLUT1 expression in B16 melanoma cells **A.** Quantitative RT-PCR and **B.** western blot analysis of GLUT1 expression in B16 melanoma cells after stable transfection with an expression vector containing GLUT1 shRNA (shRNA1 or shRNA2). B16 cells transfected with the empty vector served as control (ctrl.). **C.** GLUT1 immunohistochemical staining of cell pellets of control and GLUT1 shRNA cell clones. **D.** Glucose uptake, **E.** glucose consumption and **F.** lactate secretion of GLUT1 suppressed and control cell clones. (*p < 0.05 compared to control).

To gain insight into the functional role of increased GLUT1 in melanoma cells, we inhibited GLUT1 expression in the murine melanoma cell line B16 by stable transfection with a shRNA expression vector containing the sequence of GLUT1 siRNA (GLUT1_shRNA1 and GLUT1_shRNA2). B16 cells transfected with the empty vector served as control. Quantitative RT-PCR and western blot analysis revealed a strong downregulation of GLUT1 expression in GLUT1*_*shRNA compared to control cell clones (Figure 2A, 2B). Immunohistochemical analysis showed strong membranous GLUT1 staining in control cells, which almost completely disappeared in GLUT1 shRNA transfected cell clones (Figure 2C). GLUT1 suppressed cells revealed significantly reduced glucose uptake and consumption compared to control cells (Figure 2D, 2E). In line with this, lactate secretion was reduced in GLUT1 suppressed cells (Figure 2F). Together, these data indicate that GLUT1 is a rate-limiting factor for the glucose transport and glycolysis in melanoma cells *in vitro*.

### Effect of GLUT1 inhibition on B16 melanoma cells *in vitro*

**Figure 3:**
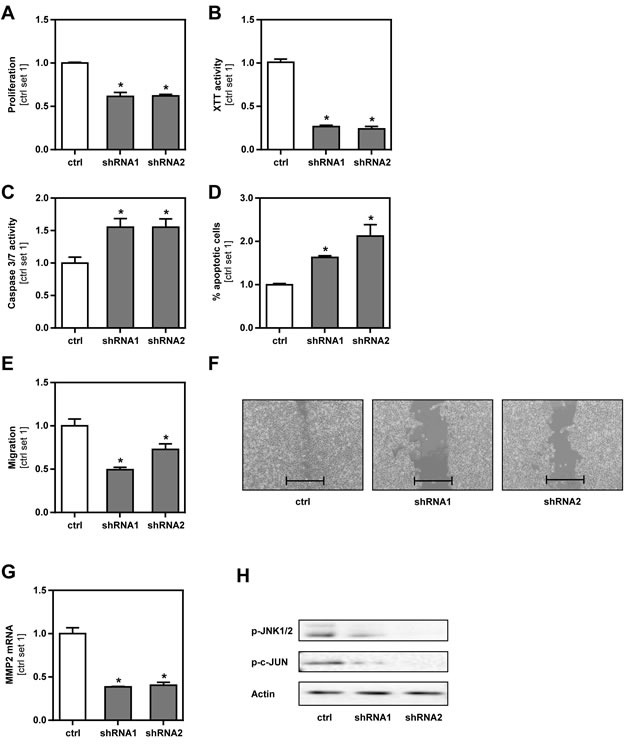
Effect of GLUT1 inhibition on B16 melanoma cells *in vitro* **A.** Proliferation (as determined by cell counting), **B.** mitochondrial activity (XTT assay), **C.** caspase 3/7 activity assay and **D.** proportion of apoptotic cells (Annexin V-FITC FACS analysis) of GLUT1 suppressed and control cells. **E.** Analysis of migratory activity with Boyden chamber assays. **F.** Analysis of cell migration in time-lapse scratch assays (bar depicts the original width of the scratch). **G.** MMP2 mRNA expression levels of GLUT1 suppressed clones compared to control cells. **H.** Western blot analysis of phosphorylated JNK1/2 (Thr183/Tyr185) and c-JUN (Ser73) in GLUT1 suppressed and control cells. (*p < 0.05 compared to control).

**Figure 4:**
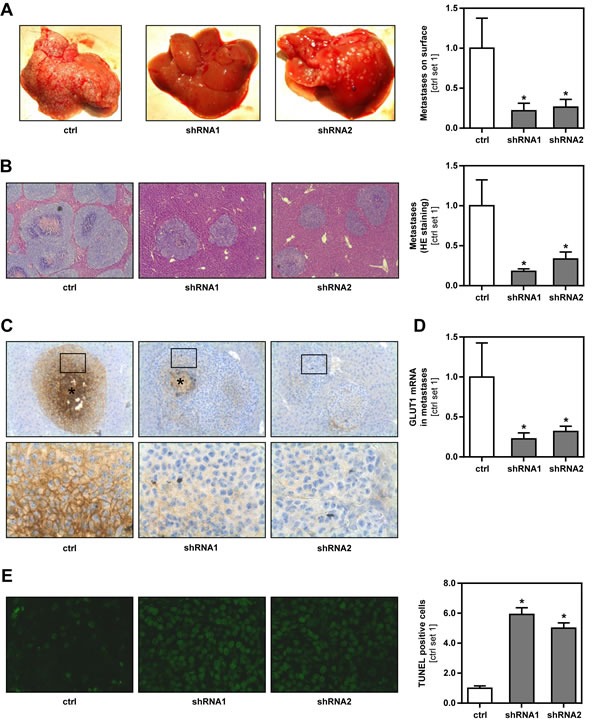
Effect of GLUT1 inhibition on hepatic metastasis of B16 melanoma cells *in vivo* Hepatic metastasis of GLUT1 suppressed and control B16 cell clones were analyzed in a syngeneic model in Bl6/N mice. **A.** Representative images of macroscopically visible metastases on the liver surface (left panel). Bar graphs depicting the number of metastases derived from GLUT1 suppressed and control cell clones on the liver surface (control set 1) (right panel). **B.** Representative images of HE stained hepatic tissue sections showing intrahepatic metastases (left panel). Bar graphs depicting the number of intrahepatic metastases formed by GLUT1 suppressed and control cell clones (control set 1) (right panel). **C.** Immunohistochemical GLUT1 staining of hepatic metastases (*: central necrosis). Squares depict areas which are shown as higher-magnification field in the lower panel. **D.** quantitative RT-PCR analysis of GLUT1 mRNA expression in hepatic metastases. **E.** TUNEL staining of hepatic metastases derived from GLUT1 suppressed and control cell clones (left panel). Bar graphs depicting the number of TUNEL positive cells (control set 1) (right panel). (*p < 0.05 compared to control).

To further characterize the role of GLUT1 in melanoma cells, we performed functional *in vitro* assays with GLUT1 suppressed and control B16 cells. All formed a homogenous cell layer and appeared similar in microscopical analysis (Suppl.Figure 1). However, GLUT1 suppression significantly reduced cell growth (Figure 3A, Suppl.Figure 2) and impaired mitochondrial activity as analyzed by XTT assay (Figure 3B). In addition, GLUT1 suppressed B16 cells had significantly higher caspase 3/7 activity (Figure 3C). Annexin V-FITC FACS analysis confirmed a higher apoptosis rate in GLUT1 suppressed cells (Figure 3D, Suppl.Figure 3). Interestingly, Boyden chamber assays (Figure 3E) and time-lapse scratch assays (Figure 3F, Suppl.Figure 4) revealed significantly reduced migratory activity compared to control cells. Moreover, GLUT1 suppression in B16 cells led to a significant reduction of the expression of matrix metalloproteinase 2 (MMP2) (Figure 3G), which encodes for an enzyme involved in degradation of extra-cellular matrix proteins and tumor progression. C-Jun N-terminal kinase (JNK; MAPK8) regulates a wide range of targets in the progression and metastatic cascade of cancer cells including proliferation, migration, attachment and MMP-expression [21, 22]. Notably, western blot analysis showed reduced levels of phosphorylated JNK and c-JUN in GLUT1 suppressed cell clones (Figure 3H). In summary, these findings indicate that GLUT1 expression in malignant melanoma cells promotes their tumorigenicity *in vitro* and that this is at least in part mediated *via* enhanced JNK-activity.

### Effect of GLUT1 inhibition on hepatic metastasis of melanoma cells *in vivo*

**Figure 5:**
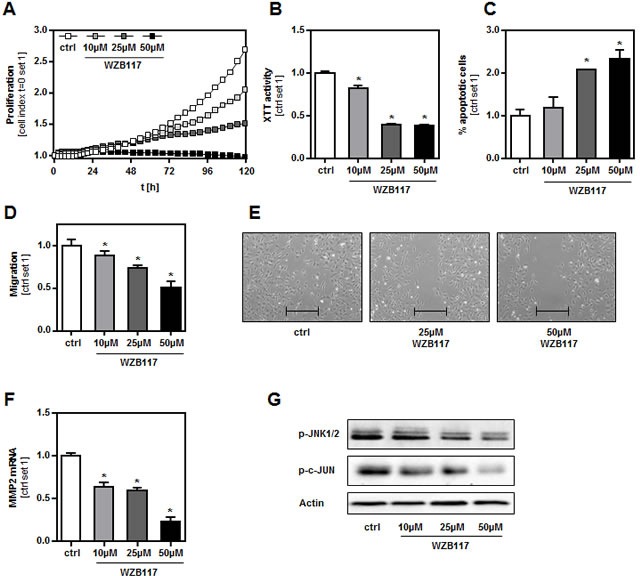
Effect of a chemical GLUT1 inhibition on human melanoma cells *in vitro* The human melanoma cell line WM3211 was treated with the chemical GLUT1 inhibitor WZB117 at the concentrations indicated. **A.** Proliferation analyzed with XCELLigence™ system, **B.** XTT activity and **C.** proportion of apoptotic cells (Annexin V-FITC FACS analysis). Migratory activity analyzed with **D.** Boyden chamber and **E.** time-lapse scratch assays (bar depicts the original width of the scratch), and **F.** MMP2 mRNA expression levels. **G.** Western blot analysis of phosphorylated JNK1/2 (Thr183/Tyr185) and c-JUN (Ser73). (*p < 0.05 compared to control).

To test the effect of GLUT1 on tumor metastasis *in vivo*, we employed an established syngeneic murine model of hepatic metastasis [23] using GLUT1 suppressed and control B16 cell clones. Macroscopic analysis showed less metastases on the liver surface of mice injected with GLUT1 suppressed cell clones (Figure 4A). Also serum levels of transaminases were significantly lower in these mice reflecting reduced hepatic tumor burden (Suppl.Figure 5). Histological analysis confirmed that GLUT1 suppressed melanoma cells formed significantly less hepatic metastases (Figure 4B). Furthermore, metastases derived from GLUT1 suppressed cell clones tended to be smaller, but differences did not reach the level of significance (Suppl.Figure 6). Quantitative RT-PCR analysis verified reduced GLUT1 mRNA expression in tumors formed by B16 cell clones stably transfected with GLUT1 shRNA (Figure 4D). Immunohistochemical analysis confirmed significantly stronger, membranous GLUT1 staining in vital areas of metastases formed by control cells clones compared to GLUT1 shRNA cell clones (Figure 4C). In both, control and GLUT1 suppressed clones, staining intensity increased from the border of the metastases to the center (Figure 4C and Suppl.Figure 7). Most likely, this resulted from increased hypoxia in the center of the metastases, as GLUT1 expression is known to be induced by hypoxia [24]. In line with the *in vitro* data, TUNEL staining revealed significant more apoptosis in hepatic metastases formed by GLUT1 suppressed cells (Figure 4E). In summary, these data demonstrate that GLUT1 expression advances metastasis of melanoma cells *in vivo*.

### Effect of chemical GLUT1 inhibition on proliferation of melanoma cells *in vitro*

The strongly impaired tumorigenicity of GLUT1 suppressed cell clones compared to control cells indicated that GLUT1 inhibition is a valuable therapeutic target to inhibit growth and metastasis of malignant melanoma cells. To get further insight into the perspective of potential clinical GLUT1 inhibitory strategies, we applied WZB117, a new, highly specific small-molecule inhibitor of GLUT1 [25], to the human melanoma cell lines WM3211, Mel-Im and SbCl2 *in vitro*. In all cell lines, incubation with WZB117 led to a dose-dependent reduction of glucose consumption and lactate secretion without affecting LDH levels in the supernatant (Suppl.Figure 8A-8C). Furthermore, pharmacological GLUT1 inhibition with WZB117 reduced proliferation (Figure 5A, Suppl.Figure 9A) and XTT activity (Figure 5B, Suppl.Figure 9B) of human melanoma cells *in vitro*. Conversely, the proportion of apoptotic cells was increased when cells were treated with WZB117 (Figure 5C, Suppl.Figure 9C). In addition, WZB117 decreased the migratory activity of human melanoma cells as shown by Boyden chamber and time-lapse scratch assays (Figure 5D, 5E, Suppl.Figure 10A, 10B). WZB117 treatment also led to a significantly reduced MMP2 expression (Figure 5F, Suppl.Figure 10C). Western blot analysis revealed decreased amounts of phosphorylated JNK and c-JUN in melanoma cells treated with WZB117 (Figure 5G, Suppl.Figure 10D). Importantly, incubation of primary human hepatocytes with WZB117 in even higher doses than applied in melanoma cells did not decrease viability or glucose uptake (data not shown). In summary, pharmacological GLUT1 inhibition in human melanoma cells revealed comparable anti-tumorigenic effects as observed in murine B16 melanoma cells with shRNA-suppressed GLUT1 expression.

## DISCUSSION

Malignant melanomas have unique tumor biology and unpredictable patterns of metastasis. The aim of this study was to analyze the expression and function of GLUT1 in the progression of this highly aggressive form of skin cancer.

Increased GLUT1 expression has been demonstrated in several types of human tumors compared to non-cancerous tissues [7, 8, 24, 26-28]. However, most of the published reports studied epithelial neoplasms, whereas nevi and malignant melanomas arise from melanocytes, which originate from the neural crest, and it is well known that melanocytes and derived cancerous cells have unique metabolic pathways that may interfere with glycolysis.

Previous studies of GLUT1 expression in melanoma have shown inconclusive and in part opposite results. Applying western blot technique, Wachsberger *et al.* observed a strong variation in GLUT1 levels in primary melanoma tissues but did not compare expression levels with non-malignant tissues [17]. Baer *et al.* and Parente *et al*. reported less frequent GLUT1 in melanomas as compared to melanocytic nevi and the majority of Spitz nevi [18, 29]. However, they analyzed only small numbers of specimens. In contrast, a recent study by Slominski *et al*. showed significantly higher levels of GLUT1 in 75 melanomas compared to 26 melanocytic nevi [19]. Here, we confirmed the increased GLUT1 expression in 78 melanomas compared with 128 nevi. Most importantly, we found that GLUT1 expression in primary melanomas was an indicator for progression free- and overall survival. Previous studies reported various associations between GLUT1 expression and tumor aggressiveness and a poor prognosis in a number of other malignancies including pancreatic, pulmonary, colorectal, hepatocellular, ovarian and squamous cell carcinoma [27, 28, 30-32]. One explanation for the correlation between GLUT1 expression and a poor prognosis in melanoma and other types of cancer may be an increased utilization of energy and faster cell growth. Indeed, we and others have shown in the past that GLUT1 expression levels in various cancer cell types correlate with induced glycolytic activity and increased proliferation [14, 26, 33, 34]. Here, we show that also in melanoma cells GLUT1 expression promotes glucose uptake and cell growth. And also in human melanoma tissues, we found a significant correlation between GLUT1 expression and mitotic activity. Thus, induction of the growth of the primary tumor appears as one mechanism by which GLUT1 expression induces melanoma progression and metastasis.

In addition, we identified further mechanisms by which GLUT1 levels induce metastasis of melanoma cells. Matrix metalloproteinases (MMPs) are degradative enzymes that play an important role in all aspects of tumor progression by enhancing tumor-induced angiogenesis. Furthermore, MMPs destroy the local tissue architecture and basement membranes allowing tumor invasion and metastasis [35]. Here, we demonstrate that inhibition of GLUT1 inhibition lowers MMP2 expression in melanoma cells. MMP2 has been shown to be associated with melanoma progression [36, 37]. Moreover, we newly found that GLUT1 inhibits apoptosis of melanoma cells *in vitro* and *in vivo*. Hereby, we identified one further mechanism by which GLUT1 promotes melanoma growth and metastasis. JNK regulates a wide range of targets in the metastatic cascade [21]. Also in melanoma, this pathway is a critical regulator of tumor progression [22, 38]. Noteworthy, we observed that GLUT1 inhibition caused a significant inhibition of JNK-activity. To the best of our knowledge, impairment of glycolysis or components of the glycolytic cascade, respectively, has not been connected with MAPK-activity. Our data indicate that the protumorigenic effects of GLUT1 in melanoma are at least in part mediated *via* JNK-activation.

In the light of the direct impact of GLUT1 expression on different protumorigenic mechanisms in melanoma, it was important that we further found that melanoma metastases revealed stronger GLUT1 expression than primary human melanomas, which was in line with a previous study from Mihic-Probst *et al.* [39]. The focus of this elegant study was vasculogenic mimicry and neovascularization in human melanoma, and the authors observed an inverse correlation between blood vessel number and GLUT1 expression. Consequently, they suggested that the hypoxic microenvironment in metastases contributes to GLUT1 expression. Moreover, Lee *et al.* analyzed GLUT1 expression in pulmonary metastases of melanoma patients and found that GLUT1 staining intensity was a significant predictor of postmetastasectomy survival [40]. Together with our new findings in melanoma, these studies indicate that there may be at least two mechanisms which explain higher GLUT1 expression in melanoma metastases: (i) origin from primary tumors with already higher expression levels and (ii) additional induction by hypoxia or other environmental factors at the place of metastasis. Furthermore, these data indicate GLUT1 expression in primary tumors and melanoma metastases as potential prognostic marker or marker for therapeutic decisions, respectively. If biopsied tissue is not available, 18F-fluorodeoxyglucose positron emission tomography (FDG-PET) can be used for clinical evaluation and quantification of glycolytic phenotype and a surrogate for GLUT1 expression in malignant melanoma (metastases). Park *et al.* described a positive correlation between FDG uptake and GLUT1 expression in metastases of melanoma patients [15]. Also several other reports indicate that the expression of GLUT1 correlates with the uptake of the glucose analogue ^18^F-FDG in a variety of human tumors [41-43]. Moreover, Baudy *et al*. found that ^18^F-FDG can be a sensitive biomarker for assessing efficacy and also resistance to the BRAF inhibitor vemurafenib [44]. Mechanistically, they found that this resistance was associated with the induction of GLUT1. Other studies indicate that FDG-PET correlated with the response to further classes of chemotherapeutics in melanoma patients [45, 46]. Furthermore, Giammarioli *et al*. showed that inhibition of glucose metabolism by using glucose analogue 2-Deoxy-D-glucose acted synergistic with cisplatin against melanoma cells *in vitro* [47]. Still, to the best of our knowledge there are no studies analyzing the effect of GLUT1 inhibition in melanoma cells on the efficacy of chemotherapeutic agents and on therapy resistance, respectively. However, studies in several other cancer cells such as colon, hepatocellular, head and neck or oral squamous cell carcinoma indicate that GLUT1 inhibition may be considered an additional treatment option for resistant forms of cancers [48-50].

The therapeutic potential of GLUT1 inhibitory strategies alone or together with other therapeutic strategies in (metastatic) melanoma is yet to be determined, but our data provide additional evidence to pursue such studies. Furthermore, our findings promote GLUT1 levels in primary melanoma tissues as well as in metastases as a prognostic marker for this highly aggressive tumor.

## MATERIALS AND METHODS

### Cells and cell culture

The murine melanoma cell line B16 and the human melanoma cell lines Mel-Im, SbCl2 and WM3211 were cultured as described [23]. For some experiments, cells were treated with the small-molecule GLUT1-inhibitor WZB117 (EMD Chemicals, San Diego, CA, USA) [25] with different concentrations and time periods as indicated. The vehicle DMSO alone served as control.

### Stable transfection of B16 melanoma cells

A panel of B16 cell clones with decreased GLUT1 expression were established by stable transfection with short hairpin RNA (shRNA) plasmids containing either antisense GLUT1-shRNA and neomycin resistance (*shRNA1, shRNA2*; Sure Silencing shRNA Plasmid, Mouse Slc2a1, Qiagen, Hilden, Germany) or neomycin resistance alone (*ctrl*; Sure Silencing shRNA Plasmid, Negative Control-Neomycin, Qiagen) as described [23]. Paraffin-embedded pellets of cultured cells were generated using Shandon Cytoblock Cell Block Preparation System (Thermo Scientific, Waltham, MA, USA) according to the supplier's instructions.

### Human malignant melanoma tissue micro array (TMA)

A tissue micro array (TMA) comprising specimens from benign nevi, primary human melanoma and melanoma metastases was constructed as described [20]. Clinicopathological patient characteristics are summarized in Table 1. The median follow up for all patients was 54 months (range 1-135 months). The University of Regensburg Institutional Review Board granted approval for the project.

### Analysis of mRNA expression

Isolation of total cellular RNA from cells and tissues and reverse transcription were performed as described [51]. Quantitative real-time PCR was performed applying LightCycler technology (Roche, Mannheim, Germany) [51] and the following pairs of primers: human GLUT1 (forward: 5′-AAC TCT TCA GCC AGG GTC CAC; reverse: 5′-CAC AGT GAA GAT GAT GAA GAC) and human MMP2 (forward: 5′-GCT GGG AGC ATG GCG ATG GAT ACC-3′; reverse: 5′-GGA CAG AAG CCG TAC TTG CCA TCC-3′). Murine GLUT1 and murine MMP2 mRNA expression analyses were performed using QuantiTect Primer Assays according to the manufacturer's instructions (Qiagen). Amplification of cDNA derived from 18S rRNA (forward: 5′-AAA CGG CTA CCA CAT CCA AG-3′; reverse: 5′-CCT CCA ATG GAT CCT CGT TA-3′) was used for normalization.

### Protein analysis

Protein extraction and western blotting were performed as described [51] applying the following primary antibodies: rabbit polyclonal anti-GLUT1 (Thermo Scientific, RB-9052, 1 : 1,000), rabbit polyclonal anti-phospho-SAPK/JNK (Cell Signaling Technology, Leiden, The Netherlands; #9251, 1 : 1,000), rabbit monoclonal anti-phospho-c-JUN (Cell Signaling Technology, #3270, 1 : 1,000) and mouse monoclonal anti-Actin (ACTB; Merck Millipore, Billerica, MA, USA; MAB1501, 1 : 10,000). Goat anti-rabbit (Santa Cruz Biotechnology, Heidelberg, Germany; sc-2030, 1 : 2,500) and goat anti-mouse (Santa Cruz Biotechnology, sc-2005, 1 : 2,500) were used as secondary antibodies.

### Histological and immunohistological analyses

For immunohistochemistry and hematoxylin and eosin (HE) staining, standard 5 μm sections of formalin-ﬁxed and parafﬁn-embedded tissue blocks were used. Immunohistochemical staining was performed using the following antibodies as described [51]: anti-GLUT1 (Thermo Scientific, RB-9052, 1 : 100), anti-KI67 (Abcam, Cambridge, UK; MIB1, 1 : 50). Immunohistochemical stainings were counterstained with hematoxylin. For analysis of the TMA, GLUT1 staining intensity was scored semiquantitatively as 0, 1 or 2. The staining intensity was defined as negative (score 0), weak (score 1) and strong (score 2) with reference to the immunostaining of erythrocytes, which were scored as strong. The GLUT1 score was specified based on the staining intensity of the majority of melanoma cells. Representative examples of melanoma specimens with these three GLUT1 staining intensities are depicted in Figure 1A (right panel).

### Glucose uptake

Glucose uptake was measured by incubating cells with 2-(N-(7-Nitrobenz-2-oxa-1,3-diazol-4-yl)Amino)-2-Deoxyglucose (2-NBDG; Thermo Scientific N13195) and subsequent fluorescence-activated cell sorting (FACS) analysis as described [26].

### Proliferation and mitochondrial activity assays

Cell proliferation was measured using the xCELLigence system (Roche) according to the manufacturer's instructions. In addition, cell proliferation was determined by cell counting. Here, 1 × 10^6^ cells were seeded into T75 flasks. After 48h, cells were trypsinized and microscopically counted. Mitochondrial activity was measured applying a colorimetric XTT assay (Roche) [51].

### Caspase-3/7 activity assay, annexin V-FITC FACS analysis and TUNEL assay

Caspase-3/7 activity was analyzed using the Apo-One homogeneous caspase-3/7 assay (Promega, Mannheim, Germany) as described [52]. For detection of apoptosis, cells were stained simultaneously with FITC-conjugated Annexin V and propidium iodide (both Promokine, Heidelberg, Germany) and analyzed by flow cytometry as described [52]. Flowing Software™ version 2.51 (Perttu Terho, Turku, Finland) was used for FACS data analysis. Terminal deoxynucleotidyl transferase-mediated dUTP nick end labeling staining (TUNEL, Promega) was performed according to the manufacturer's instructions. For statistical analysis, TUNEL positive cells were counted in 4 representative sections per sample.

### Analysis of migratiory activity of melanoma cells *in vitro*

Migratory activity of melanoma cells was quantified using Cultrex 96 Well Cell Migration assay (Trevigen, Gaithersburg, MD, USA) as described [26]. Further, cell migration was assessed applying time-lapse scratch assays (‘wound-healing-assay’) as described [53]. Briefly, nearly confluent cell monolayers were scratched and migration of cells into the wounded area was monitored throughout a 24h time window.

### *In vivo* metastasis assay

To determine the metastatic potential of murine B16 melanoma cells *in vivo,* a mouse model of hepatic metastasis was used [23]. Monodispersed tumor cells (1 × 10^6^ in 50μl) were injected into the spleen of syngeneic Bl/6N mice (*n* = 7 per group; age: 16 weeks, mean bodyweight: 24g; Charles River Laboratories, Sulzfeld, Germany). After 11 days, mice were sacrificed and the livers were processed for further analyses.

All experimental protocols were approved by the Committee on Animal Health and Care of the local government, and conformed to international guidelines on the ethical use of animals.

### Statistical analysis and image acquisition tools

Results are expressed as mean ± SEM. Comparison between groups was made using ordinary one-way ANOVA. A *p*-value < 0.05 was considered statistically significant. Contingency table analysis and two-sided Fisher's exact tests were used to study the statistical association between categorical clinicopathological and immunohistochemical variables. Retrospective overall survival and progression-free survival curves comparing patients with and without any of the variables were calculated using the Kaplan-Meier method, with significance evaluated using log-rank statistics. For the progression-free survival analysis, patients were censored at the time of their last tumor-free clinical follow-up appointment. For the overall survival analysis, patients were censored at the time of their last clinical follow-up appointment. Median overall survival time for censored patients was 63.5 months (range: 3-135 months). Calculations were performed by using the GraphPad Prism Software version 6.01 (GraphPad Software, San Diego, CA, USA) and SPSS Software version 21.0 (SPSS, Chicago, IL, USA).

Microscopical images were taken using an Olympus™ CKX41 microscope with the ALTRA 20 Soft Imaging System™ and Cell^A^ software version 2.6 (Olympus Soft Imaging Solutions GmbH, Münster, Germany). Images were processed using IrfanView™ software version 4.36 (Irfan Skiljan, Jajce, Bosnia).

## SUPPLEMENTARY MATERIAL FIGURES



## References

[1] Morton DL, Thompson JF, Cochran AJ, Mozzillo N, Nieweg OE, Roses DF, Hoekstra HJ, Karakousis CP, Puleo CA, Coventry BJ, Kashani-Sabet M, Smithers BM, Paul E, Kraybill WG, McKinnon JG, Wang HJ (2014). Final trial report of sentinel-node biopsy versus nodal observation in melanoma. N Engl J Med.

[2] Balch CM, Soong SJ, Gershenwald JE, Thompson JF, Reintgen DS, Cascinelli N, Urist M, McMasters KM, Ross MI, Kirkwood JM, Atkins MB, Thompson JA, Coit DG, Byrd D, Desmond R, Zhang Y (2001). Prognostic factors analysis of 17,600 melanoma patients: validation of the American Joint Committee on Cancer melanoma staging system. J Clin Oncol.

[3] Leiter U, Eigentler T, Garbe C (2014). Epidemiology of skin cancer. Adv Exp Med Biol.

[4] Tronnier M, Mitteldorf C (2014). Treating advanced melanoma: current insights and opportunities. Cancer Manag Res.

[5] Kim JW, Dang CV (2006). Cancer's molecular sweet tooth and the Warburg effect. Cancer Res.

[6] Brahimi-Horn MC, Chiche J, Pouyssegur J (2007). Hypoxia signalling controls metabolic demand. Curr Opin Cell Biol.

[7] Airley RE, Mobasheri A (2007). Hypoxic regulation of glucose transport, anaerobic metabolism and angiogenesis in cancer: novel pathways and targets for anticancer therapeutics. Chemotherapy.

[8] Medina RA, Owen GI (2002). Glucose transporters: expression, regulation and cancer. Biol Res.

[9] Amann T, Hellerbrand C (2009). GLUT1 as a therapeutic target in hepatocellular carcinoma. Expert opinion on therapeutic targets.

[10] Cooper R, Sarioglu S, Sokmen S, Fuzun M, Kupelioglu A, Valentine H, Gorken IB, Airley R, West C (2003). Glucose transporter-1 (GLUT-1): a potential marker of prognosis in rectal carcinoma?. Br J Cancer.

[11] Oliver RJ, Woodwards RT, Sloan P, Thakker NS, Stratford IJ, Airley RE (2004). Prognostic value of facilitative glucose transporter Glut-1 in oral squamous cell carcinomas treated by surgical resection; results of EORTC Translational Research Fund studies. Eur J Cancer.

[12] Younes M, Lechago LV, Lechago J (1996). Overexpression of the human erythrocyte glucose transporter occurs as a late event in human colorectal carcinogenesis and is associated with an increased incidence of lymph node metastases. Clin Cancer Res.

[13] Haber RS, Rathan A, Weiser KR, Pritsker A, Itzkowitz SH, Bodian C, Slater G, Weiss A, Burstein DE (1998). GLUT1 glucose transporter expression in colorectal carcinoma: a marker for poor prognosis. Cancer.

[14] Nagarajah J, Grabellus F, Schmid K, Bockisch A, Sheu S (2011). GLUT1 expression, tumor proliferation, and iodine/FDG-uptake in thyroid cancer with emphasis on poorly-differentiated thyroid carcinoma. Eur J Nucl Med Mol Imaging.

[15] Park SG, Lee JH, Lee WA, Han KM (2012). Biologic correlation between glucose transporters, hexokinase-II, Ki-67 and FDG uptake in malignant melanoma. Nucl Med Biol.

[16] Yamada K, Brink I (2005). Factors influencing [F-18]. J Dermatol.

[17] Wachsberger PR, Gressen EL, Bhala A, Bobyock SB, Storck C, Coss RA, Berd D, Leeper DB (2002). Variability in glucose transporter-1 levels and hexokinase activity in human melanoma. Melanoma Res.

[18] Baer SC, Casaubon L, Younes M (1997). Expression of the human erythrocyte glucose transporter Glut1 in cutaneous neoplasia. J Am Acad Dermatol.

[19] Slominski A, Kim TK, Brozyna AA, Janjetovic Z, Brooks DL, Schwab LP, Skobowiat C, Jozwicki W, Seagroves TN (2014). The role of melanogenesis in regulation of melanoma behavior: Melanogenesis leads to stimulation of HIF-1alpha expression and HIF-dependent attendant pathways. Arch Biochem Biophys.

[20] Massoumi R, Kuphal S, Hellerbrand C, Haas B, Wild P, Spruss T, Pfeifer A, Fassler R, Bosserhoff AK (2009). Down-regulation of CYLD expression by Snail promotes tumor progression in malignant melanoma. J Exp Med.

[21] Ebelt ND, Cantrell MA, Van Den Berg CL (2013). c-Jun N-Terminal Kinases Mediate a Wide Range of Targets in the Metastatic Cascade. Genes Cancer.

[22] Kappelmann M, Bosserhoff A, Kuphal S (2014). AP-1/c-Jun transcription factors: regulation and function in malignant melanoma. Eur J Cell Biol.

[23] Schmidt J, Riechers A, Stoll R, Amann T, Fink F, Spruss T, Gronwald W, Konig B, Hellerbrand C, Bosserhoff AK (2012). Targeting melanoma metastasis and immunosuppression with a new mode of melanoma inhibitory activity (MIA) protein inhibition. PLoS One.

[24] Amann T, Hellerbrand C (2009). GLUT1 as a therapeutic target in hepatocellular carcinoma. Expert Opin Ther Targets.

[25] Liu Y, Cao Y, Zhang W, Bergmeier S, Qian Y, Akbar H, Colvin R, Ding J, Tong L, Wu S, Hines J, Chen X (2012). A small-molecule inhibitor of glucose transporter 1 downregulates glycolysis, induces cell-cycle arrest, and inhibits cancer cell growth *in vitro* and *in vivo*. Mol Cancer Ther.

[26] Amann T, Maegdefrau U, Hartmann A, Agaimy A, Marienhagen J, Weiss TS, Stoeltzing O, Warnecke C, Scholmerich J, Oefner PJ, Kreutz M, Bosserhoff AK, Hellerbrand C (2009). GLUT1 expression is increased in hepatocellular carcinoma and promotes tumorigenesis. Am J Pathol.

[27] Ito T, Noguchi Y, Satoh S, Hayashi H, Inayama Y, Kitamura H (1998). Expression of facilitative glucose transporter isoforms in lung carcinomas: its relation to histologic type, differentiation grade, and tumor stage. Mod Pathol.

[28] Jun YJ, Jang SM, Han HL, Lee KH, Jang KS, Paik SS (2011). Clinicopathologic significance of GLUT1 expression and its correlation with Apaf-1 in colorectal adenocarcinomas. World J Gastroenterol.

[29] Parente P, Coli A, Massi G, Mangoni A, Fabrizi MM, Bigotti G (2008). Immunohistochemical expression of the glucose transporters Glut-1 and Glut-3 in human malignant melanomas and benign melanocytic lesions. J Exp Clin Cancer Res.

[30] Ito H, Duxbury M, Zinner MJ, Ashley SW, Whang EE (2004). Glucose transporter-1 gene expression is associated with pancreatic cancer invasiveness and MMP-2 activity. Surgery.

[31] Kunkel M, Moergel M, Stockinger M, Jeong JH, Fritz G, Lehr HA, Whiteside TL (2007). Overexpression of GLUT-1 is associated with resistance to radiotherapy and adverse prognosis in squamous cell carcinoma of the oral cavity. Oral Oncol.

[32] Sakashita M, Aoyama N, Minami R, Maekawa S, Kuroda K, Shirasaka D, Ichihara T, Kuroda Y, Maeda S, Kasuga M (2001). Glut1 expression in T1 and T2 stage colorectal carcinomas: its relationship to clinicopathological features. Eur J Cancer.

[33] Liu T, Kishton RJ, Macintyre AN, Gerriets VA, Xiang H, Liu X, Dale Abel E, Rizzieri D, Locasale JW, Rathmell JC (2014). Glucose transporter 1-mediated glucose uptake is limiting for B-cell acute lymphoblastic leukemia anabolic metabolism and resistance to apoptosis. Cell Death Dis.

[34] Fumarola C, Caffarra C, La Monica S, Galetti M, Alfieri RR, Cavazzoni A, Galvani E, Generali D, Petronini PG, Bonelli MA (2013). Effects of sorafenib on energy metabolism in breast cancer cells: role of AMPK-mTORC1 signaling. Breast Cancer Res Treat.

[35] Kerkela E, Saarialho-Kere U (2003). Matrix metalloproteinases in tumor progression: focus on basal and squamous cell skin cancer. Exp Dermatol.

[36] Kamyab-Hesari K, Mohtasham N, Aghazadeh N, Biglarian M, Memar B, Kadeh H (2014). The expression of MMP-2 and Ki-67 in head and neck melanoma, and their correlation with clinic-pathologic indices. J Cancer Res Ther.

[37] Shaverdashvili K, Wong P, Ma J, Zhang K, Osman I, Bedogni B (2014). MT1-MMP modulates melanoma cell dissemination and metastasis through activation of MMP2 and RAC1. Pigment Cell Melanoma Res.

[38] Lopez-Bergami P, Huang C, Goydos JS, Yip D, Bar-Eli M, Herlyn M, Smalley KS, Mahale A, Eroshkin A, Aaronson S, Ronai Z (2007). Rewired ERK-JNK signaling pathways in melanoma. Cancer Cell.

[39] Mihic-Probst D, Ikenberg K, Tinguely M, Schraml P, Behnke S, Seifert B, Civenni G, Sommer L, Moch H, Dummer R (2012). Tumor cell plasticity and angiogenesis in human melanomas. PLoS One.

[40] Lee JH, Gulec SA, Kyshtoobayeva A, Sim MS, Morton DL (2009). Biological factors, tumor growth kinetics, and survival after metastasectomy for pulmonary melanoma. Ann Surg Oncol.

[41] de Geus-Oei LF, van Krieken JH, Aliredjo RP, Krabbe PF, Frielink C, Verhagen AF, Boerman OC, Oyen WJ (2007). Biological correlates of FDG uptake in non-small cell lung cancer. Lung Cancer.

[42] Yen TC, See LC, Lai CH, Yah-Huei CW, Ng KK, Ma SY, Lin WJ, Chen JT, Chen WJ, Lai CR, Hsueh S (2004). 18F-FDG uptake in squamous cell carcinoma of the cervix is correlated with glucose transporter 1 expression. J Nucl Med.

[43] Gu J, Yamamoto H, Fukunaga H, Danno K, Takemasa I, Ikeda M, Tatsumi M, Sekimoto M, Hatazawa J, Nishimura T, Monden M (2006). Correlation of GLUT-1 overexpression, tumor size, and depth of invasion with 18F-2-fluoro-2-deoxy-D-glucose uptake by positron emission tomography in colorectal cancer. Dig Dis Sci.

[44] Baudy AR, Dogan T, Flores-Mercado JE, Hoeflich KP, Su F, van Bruggen N, Williams SP (2012). FDG-PET is a good biomarker of both early response and acquired resistance in BRAFV600 mutant melanomas treated with vemurafenib and the MEK inhibitor GDC-0973. EJNMMI Res.

[45] Strobel K, Dummer R, Steinert HC, Conzett KB, Schad K, Lago MP, Soyka JD, Veit-Haibach P, Seifert B, Kalff V (2008). Chemotherapy response assessment in stage IV melanoma patients-comparison of 18F-FDG-PET/CT, CT, brain MRI, and tumormarker S-100B. Eur J Nucl Med Mol Imaging.

[46] Hofman MS, Constantinidou A, Acland K, Healy C, Harries M, O'Doherty M, Melanoma G (2007). Assessing response to chemotherapy in metastatic melanoma with FDG PET: Early experience. Nucl Med Commun.

[47] Giammarioli AM, Gambardella L, Barbati C, Pietraforte D, Tinari A, Alberton M, Gnessi L, Griffin RJ, Minetti M, Malorni W (2012). Differential effects of the glycolysis inhibitor 2-deoxy-D-glucose on the activity of pro-apoptotic agents in metastatic melanoma cells, and induction of a cytoprotective autophagic response. Int J Cancer.

[48] Liu W, Fang Y, Wang XT, Liu J, Dan X, Sun LL (2014). Overcoming 5-Fu Resistance of Colon Cells through Inhibition of Glut1 by the Specific Inhibitor WZB117. Asian Pac J Cancer Prev.

[49] Shimanishi M, Ogi K, Sogabe Y, Kaneko T, Dehari H, Miyazaki A, Hiratsuka H (2013). Silencing of GLUT-1 inhibits sensitization of oral cancer cells to cisplatin during hypoxia. J Oral Pathol Med.

[50] He C, Sun XP, Qiao H, Jiang X, Wang D, Jin X, Dong X, Wang J, Jiang H, Sun X (2012). Downregulating hypoxia-inducible factor-2alpha improves the efficacy of doxorubicin in the treatment of hepatocellular carcinoma. Cancer Sci.

[51] Hellerbrand C, Muhlbauer M, Wallner S, Schuierer M, Behrmann I, Bataille F, Weiss T, Scholmerich J, Bosserhoff AK (2006). Promoter-hypermethylation is causing functional relevant downregulation of methylthioadenosine phosphorylase (MTAP) expression in hepatocellular carcinoma. Carcinogenesis.

[52] Wobser H, Dorn C, Weiss TS, Amann T, Bollheimer C, Buttner R, Scholmerich J, Hellerbrand C (2009). Lipid accumulation in hepatocytes induces fibrogenic activation of hepatic stellate cells. Cell Res.

[53] Amann T, Bataille F, Spruss T, Muhlbauer M, Gabele E, Scholmerich J, Kiefer P, Bosserhoff AK, Hellerbrand C (2009). Activated hepatic stellate cells promote tumorigenicity of hepatocellular carcinoma. Cancer Sci.

