# Supplementary material for: Glucose transporter isoform 1 expression enhances metastasis of malignant melanoma cells
Source: Oncotarget. 2015 Jul 22;6(32):32748–60. doi: 10.18632/oncotarget.4977 (PMC4741727; doi:10.18632/oncotarget.4977)
Supplement: Supplementary file 1 [file oncotarget-06-32748-s001.pdf]

Glucose transporter isoform 1 expression enhances metastasis of malignant melanoma cells

Supplementary Material

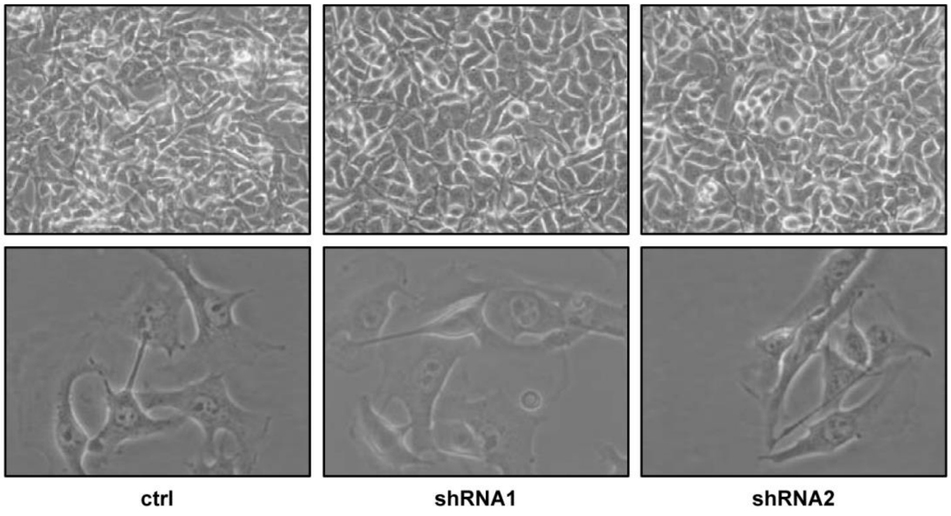

Supplementary Figure 1: Microscopical images of GLUT1 suppressed (shRNA1 and shRNA2) and control (ctrl) cell clones.

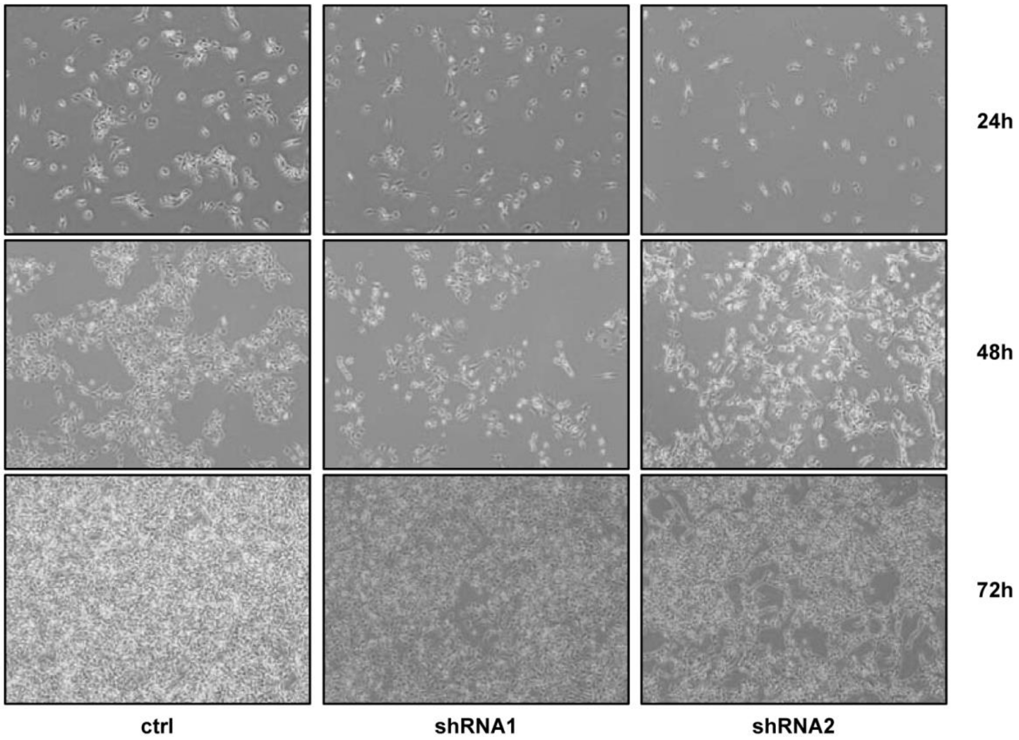

Supplementary Figure 2: Microscopical analysis of growth of GLUT1 suppressed (shRNA1 and shRNA2) and control (ctrl) cell clones after 24, 48 and 72 hours.

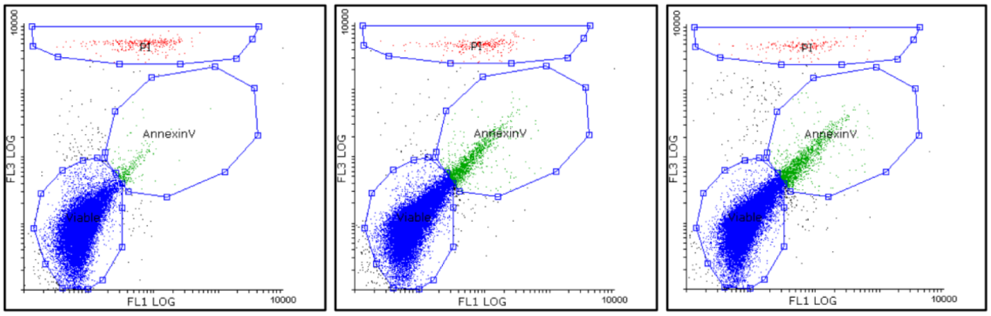

ctrl **shRNA1** **shRNA2**

Supplementary Figure 3: Flow plots of Annexin V-FITC FACS analysis of GLUT1 suppressed (shRNA1 and shRNA2) and control (ctrl) cell clones (**Fig.3D**).

Viable cells are depicted in blue, apoptotic cells in green (Annexin V positive) and dead cells in red (propidium iodide positive).

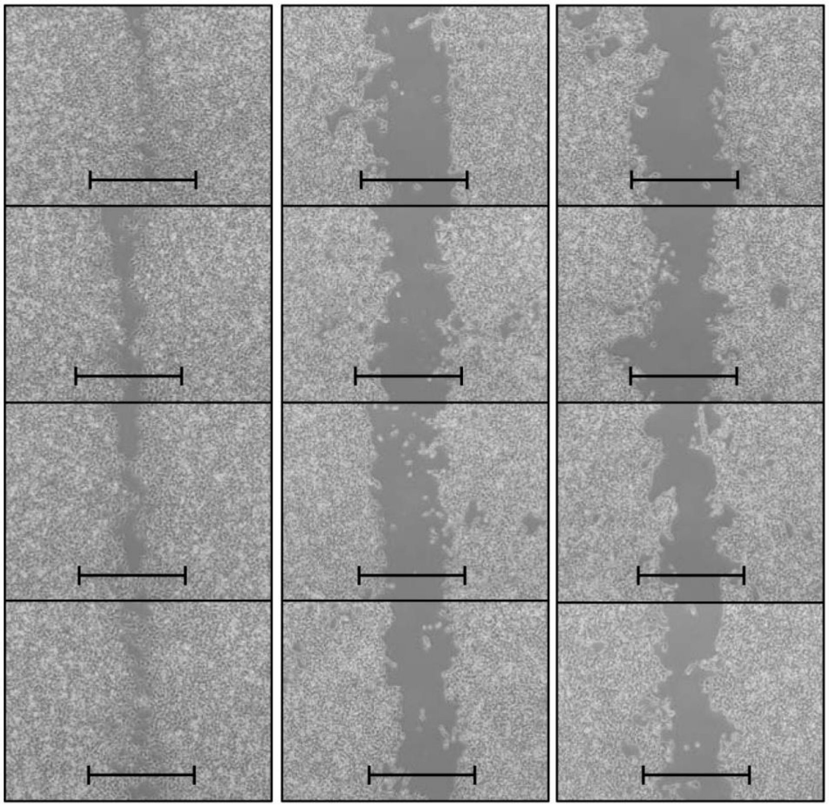

Supplementary Figure 4: Time-lapse scratch assays of control (ctrl) and GLUT1-suppressed (shRNA1, shRNA2) cell clones. Representative phase contrast images of 4 independent wound closure experiments show that after 24h, control cell clones almost closed the gap while GLUT1-suppressed clones did not fill the wounded area. Bars depict the original width of the scratch.

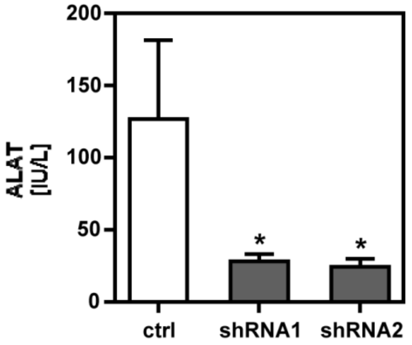

Supplementary Figure 5: Serum levels of alanine aminotransferase (ALAT) in mice bearing hepatic metastases of GLUT1 suppressed B16 cell clones (shRNA1 and shRNA2) and control (ctrl) cell clones.
(* $p < 0.05$ compared to control).

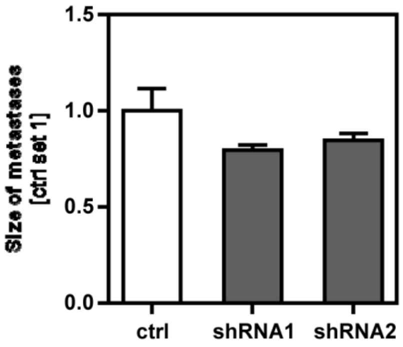

Supplementary Figure 6: Size of hepatic metastases of GLUT1 suppressed cell clones (shRNA1 and shRNA2) and control (ctrl) cell clones, as assessed in HE stained tissue slides (control set 1). No significant difference between control und shRNA groups.

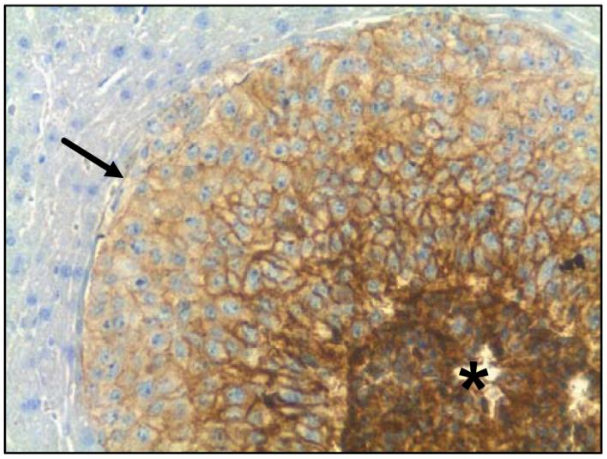

ctrl

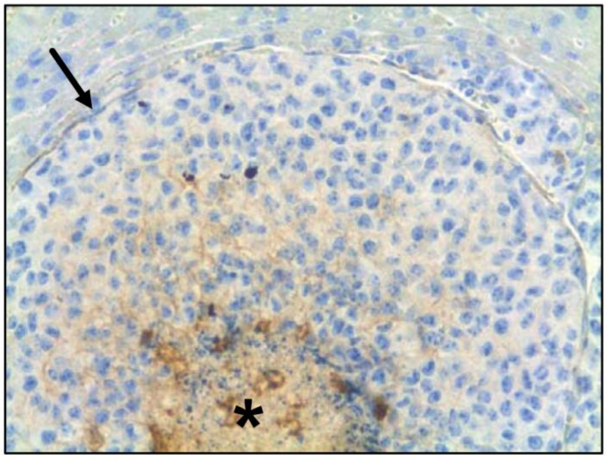

shRNA1

Supplementary Figure 7: High magnification (200x) fields of anti-GLUT1 immunohistochemical staining of hepatic tissue sections showing metastases derived from GLUT1 suppressed (shRNA1, shRNA2) and control (ctrl) cell clones. Staining intensity increases from the border of the metastases (→) towards the center. In the middle of the metastases appears unspecific staining of central necrosis (*).

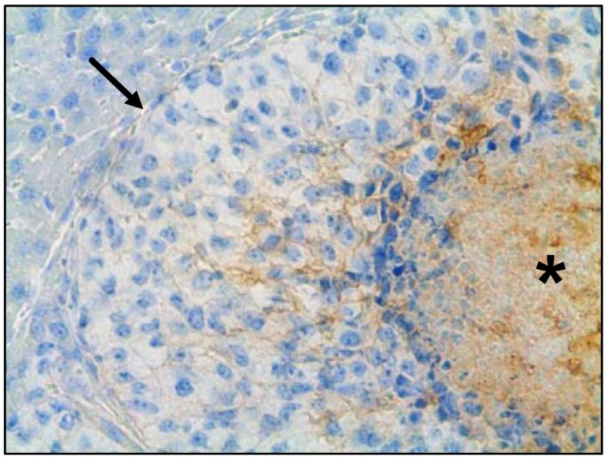

shRNA2

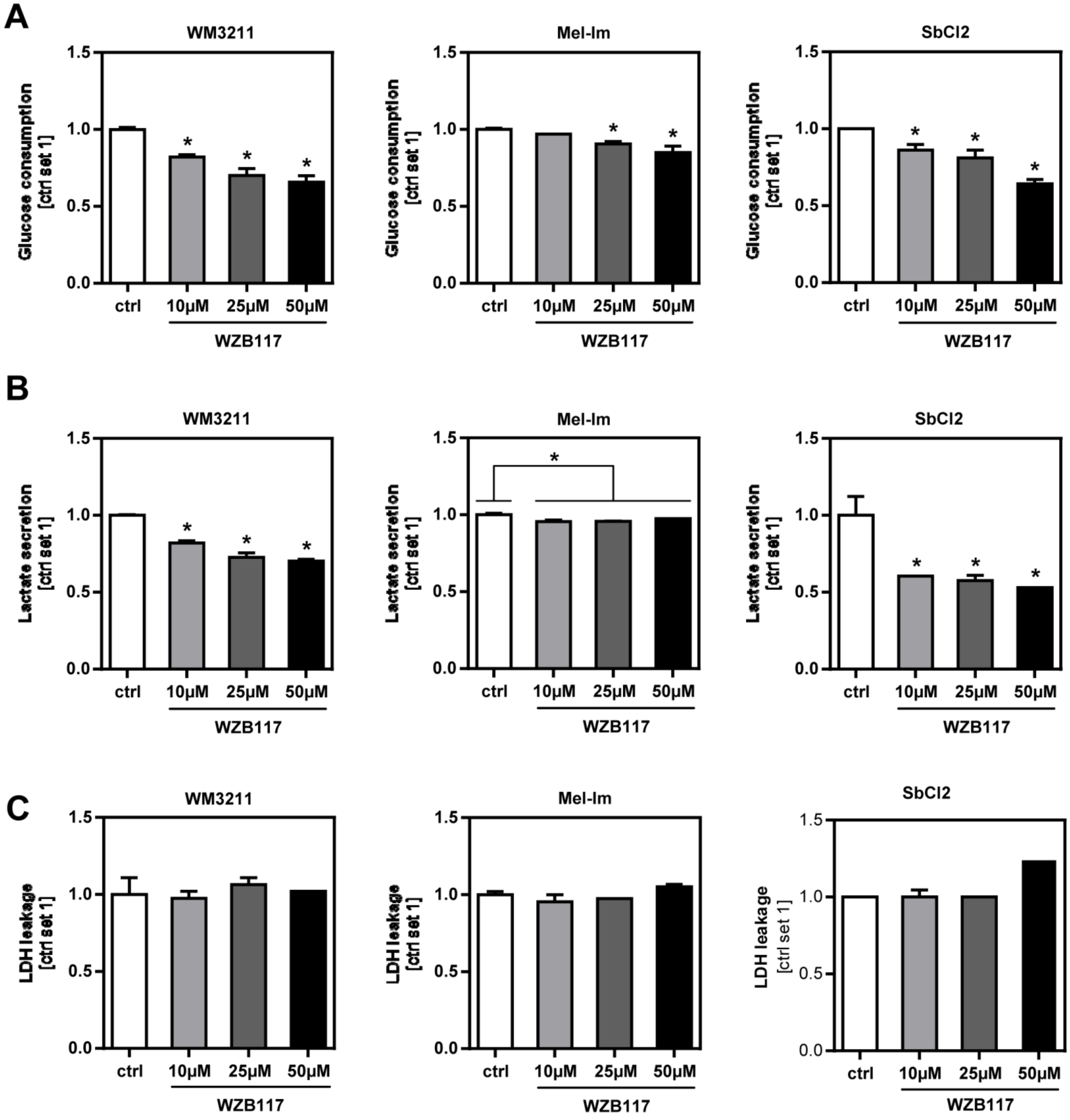

Supplementary Figure 8: (A) Glucose consumption, **(B)** lactate secretion and **(C)** lactate dehydrogenase (LDH) levels in supernatants of human melanoma cell lines WM3211, Mel-Im and SbCl2 treated with the GLUT1 inhibitor WZB117 at different concentrations. (* $p < 0.05$ compared to control).

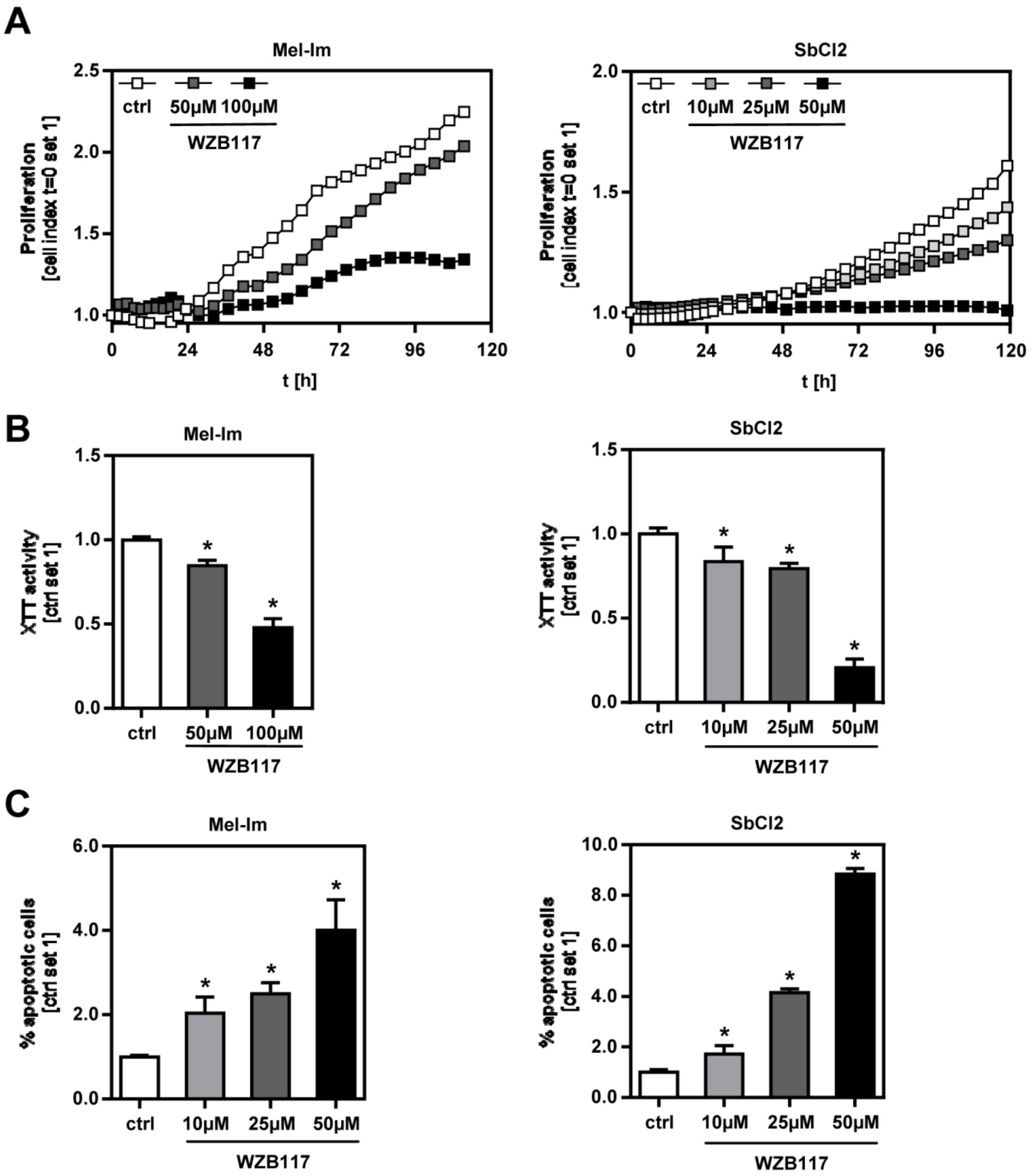

Supplementary Figure 9: (A) Proliferation (analyzed using XCELLigence™ system), **(B)** mitochondrial activity (XTT assay) and **(C)** percentage of apoptotic cells (as determined by Annexin V-FITC FACS analysis) of human melanoma cell lines Mel-Im and SbCl2 treated with the GLUT1 inhibitor WZB117. (* $p < 0.05$ compared to control).

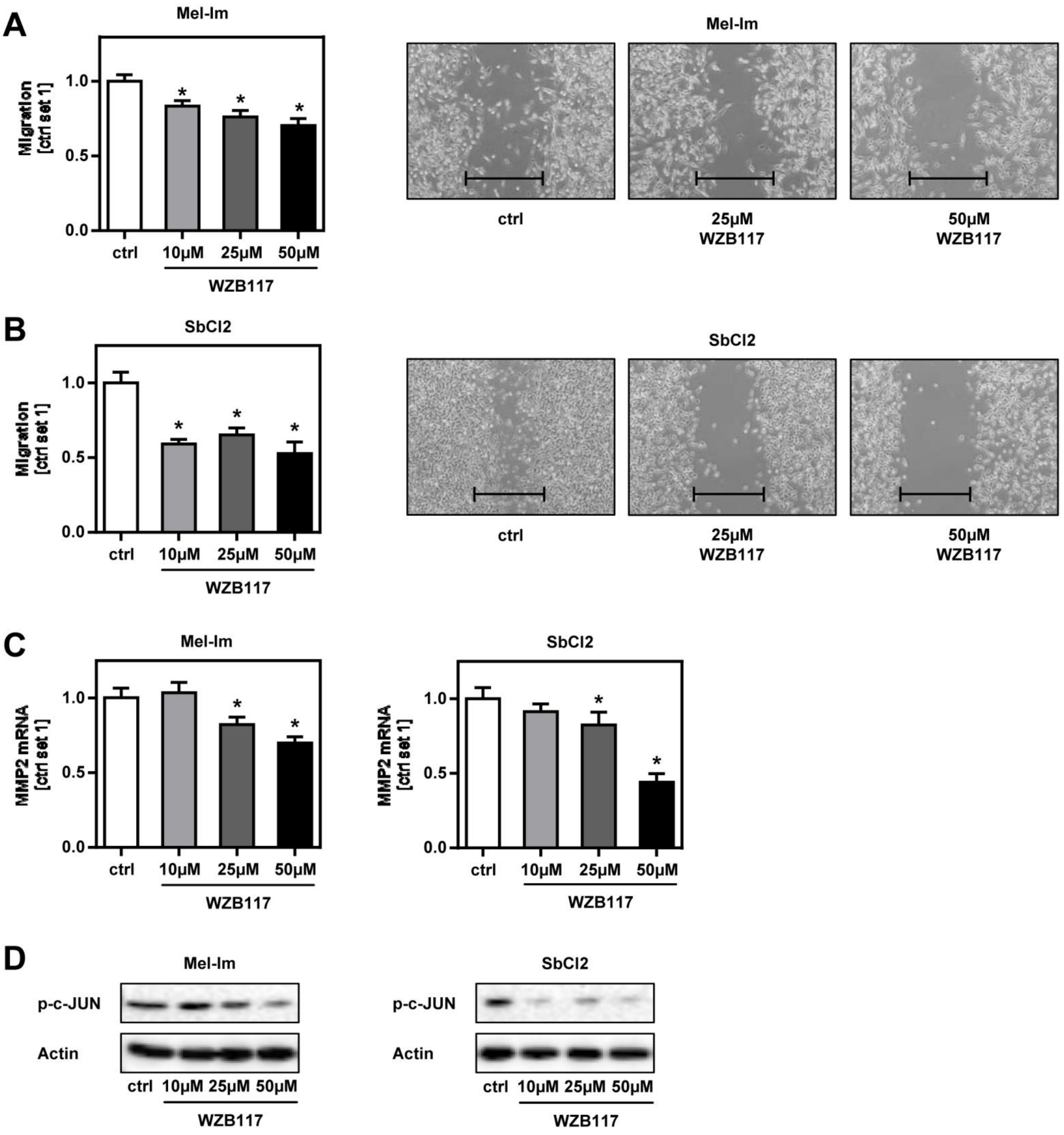

Supplementary Figure 10: The human melanoma cell lines Mel-Im and SbCl2 were treated with the chemical GLUT1 inhibitor WZB117 at the concentrations indicated. **(A, B)** Migratory actvity (as determined by Boyden chamber assays) and time-lapse scratch assay (bar depicts the original width of the scratch) of Mel-Im and SbCl2 cells. **(C)** Quantitative RT-PCR analysis of MMP2 mRNA expression. **(D)** Western blot analysis of phosphorylated c-JUN (Ser73) in Mel-Im and SbCl2 cells treated with GLUT1 inhibitor.
(* $p < 0.05$ compared to control).
